# Trends in human parainfluenza virus in Scotland before and after the peak of the COVID-19 pandemic, January 2017 to October 2023

**DOI:** 10.2807/1560-7917.ES.2025.30.2.2400147

**Published:** 2025-01-16

**Authors:** Tonje Sande Laird, Mark Hamilton, Naoma William, Shivani Karanwal, Kimberly Marsh, Josie Evans

**Affiliations:** 1 Respiratory Team, Clinical and Protecting Health Directorate, Public Health Scotland, Glasgow, United Kingdom

**Keywords:** Human parainfluenza viruses, respiratory infections, COVID-19 pandemic, surveillance, epidemiology

## Abstract

**Background:**

Human parainfluenza viruses (HPIV) commonly cause upper respiratory tract infections, with potential for severe lower respiratory complications. Understanding seasonal increases informs strategies to prevent HPIV spreading.

**Aim:**

We examined the impact of COVID-19 on HPIV epidemiological and clinical patterns in Scotland using non-sentinel and sentinel surveillance data.

**Methods:**

Information on HPIV swab positivity (January 2017–October 2023) and demographic data was obtained from the Electronic Communication of Surveillance in Scotland (ECOSS) non-sentinel surveillance sources (laboratory-based data from hospital and community) and the Community Acute Respiratory Infection (CARI) sentinel surveillance programme (enhanced surveillance and symptom data).

**Results:**

In 2020 during early COVID-19 waves, HPIV detection decreased aligning with lockdowns and preventive measures. In summer 2021, HPIV positivity increased, with HPIV-3 possibly reverting to pre-pandemic seasonality, but HPIV-1 not yet re-establishing alternate-year peaks. Most positive results from non-sentinel sources came from hospital tests. Sentinel surveillance (CARI) complemented non-sentinel data, offering community-level insights. There was no significant difference in CARI swab positivity by sex in any age group. Consistent with historical trends, children under five years exhibited highest test positivity: 9.3% (95% CI: 7.6–11.2) in females and 8.5% (95% CI 7.0–10.2) in males.

**Conclusion:**

The COVID-19 pandemic impacted HPIV detection in Scotland. The decline during the pandemic peak and subsequent partial resurgence underscores the complex interplay between viral epidemiology and public health measures. Combining diverse surveillance systems provides a comprehensive understanding of HPIV dynamics. Insights into age-specific and symptom-associated patterns contribute to understanding HPIV epidemiology and refining public health strategies.

**Key public health message**

**What did you want to address in this study and why?**

Human parainfluenza viruses (HPIV) often cause mild colds, but they can also lead to serious lung problems. This study looked at how the COVID-19 pandemic affected the circulation levels of these viruses. Understanding this can help experts devise strategies to stop different respiratory viruses from spreading.

**What have we learnt from this study?**

Cases of a common cold virus, HPIV, decreased during 2020 when COVID-19 cases peaked, but increased again in summer 2021. This shows how circulation levels of viruses are affected by public health measures. Integrating non-sentinel (ECOSS) and sentinel (CARI) surveillance data offered unique and valuable insights into these changes in the population.

**What are the implications of your findings for public health?**

The study emphasises how by combining various surveillance methods, we can gain deeper insights into the circulation levels of different viruses. This knowledge informs the development of strategies to prevent and treat infections more effectively, for example by raising awareness of these viruses, collaborating to minimise their impact, implementing measures to halt their spread, and investing in research to develop vaccines.

## Introduction

Human parainfluenza viruses (HPIV) are one of the most common causes of mild upper respiratory tract infections (e.g. the common cold) [1]. However, they can also lead to more severe lower respiratory tract complications, especially in young children, elderly people and those with weakened immune systems [1,2]. There are four types of HPIV (1–4) and two subtypes (4a and 4b) [3]. The epidemiological characteristics of the different HPIV types vary between different parts of the world depending on, among other factors, climate, population density and healthcare infrastructure. Prior to the COVID-19 pandemic, in the United Kingdom (UK) and the rest of Europe, North America and Asia (temperate regions), HPIV-1 and HPIV-2 were often associated with biennial autumn outbreaks, while HPIV-3 had a more consistent annual pattern peaking in the spring and early summer [4-6]. Human parainfluenza virus 4 has been more commonly reported since 2009, peaking in the last quarter of the year [4,7]. In tropical regions, the different HPIV types may circulate year-round, with less pronounced seasonality but minor peaks, typically during the rainy season [8]. There is evidence from sentinel and non-sentinel surveillance sources in South Korea and the United States that patterns of HPIV seasonality may have been disrupted by the COVID-19 pandemic, but this needs confirmation in other countries [9,10].

In Scotland, laboratory-based surveillance of HPIV has been carried out passively since 2008 by monitoring test results that come in through routine non-sentinel sources (the Electronic Communication of Surveillance in Scotland (ECOSS) system) [11]. Although there was also a general practitioner (GP) sentinel swabbing programme for influenza at the same time, this was limited to 200 to 250 swabs per year, of which fewer than 10% were positive for parainfluenza. In 2022, the World Health Organization (WHO) updated their guidance on severe acute respiratory syndrome coronavirus 2 (SARS-CoV-2) and influenza virologic and genomic surveillance [12], replacing two previous documents on this area [13,14]. The new guidance was based on lessons learnt as well as new evidence related to COVID-19. This resulted in a strengthening of respiratory infection surveillance in Scotland in general which has generated more comprehensive data on HPIV [15]. An important element of this was the Community Acute Respiratory Infection (CARI) sentinel surveillance programme, established in preparation for the 2021/22 winter season [16].

The aim of this study was to examine the HPIV epidemiological and clinical patterns in Scotland before and after the COVID-19 pandemic, using both non-sentinel and sentinel surveillance data.

## Methods

A combination of non-sentinel and sentinel surveillance data were used.

### Non-sentinel surveillance

For non-sentinel surveillance data we used the ECOSS system [11]. This holds all positive microbiology laboratory specimen results from community and hospital testing of patients across Scotland, with a patient identifier that can be linked to basic demographic details (e.g. age and sex). A recognised limitation of the system is that the indication for testing is unknown. Numbers of positive test results for HPIV that were either undertaken in hospitals or in the community (mainly by GPs) between 1 January 2017 and 1 October 2023 were retrieved retrospectively from ECOSS on 6 February 2024. These dates were chosen to cover the time before and after the peak of the COVID-19 pandemic. Age and sex of patient, location of test (hospital or community-based) and HPIV type were also available.

During the pandemic, public health guidance and government policies required patients with acute respiratory infection (ARI) to attend community-based COVID-19 Clinical Assessment Centres (CACs) instead of their GP practice. These were set up early on during the pandemic, and were not fully closed until April 2022. Therefore community-based GP testing for HPIV was less common during this period.

### Sentinel surveillance

From 2010, a GP sentinel swabbing programme for influenza was in place, whereby a small number of GPs were asked to swab patients with influenza-like illness or acute respiratory infection (but there was no specific case definition). A multicomponent programme was established in April 2020 for enhanced surveillance of COVID-19. In November 2021, this was superseded by the CARI sentinel surveillance programme set up In Scotland [16], generating surveillance data for HPIV.

In the CARI surveillance programme, sentinel GP practices across Scotland recruit and swab patients with symptoms of ARI. These samples are then tested for 10 respiratory pathogens by multiplex PCR (PCR) testing, including HPIV, influenza type A and B viruses, SARS-CoV-2, seasonal coronavirus, human metapneumovirus, respiratory syncytial virus (RSV), rhinovirus, adenovirus and *Mycoplasma pneumoniae*.

The number of sentinel GP practices in the CARI surveillance programme increased from around 40 in November 2021 to 164 in October 2023. General practitioners whose practices participate in the CARI surveillance programme are asked to consider testing all patients who meet the specific case definition of sudden onset of at least one of the following symptoms: cough, sore throat, shortness of breath or coryza, along with a clinician's judgement that the illness is due to an infection. In practice, this results in a convenience sample of patients that GPs have time to recruit, and who are suitable and agree to being tested. Enhanced surveillance data, including a symptoms checklist, are collected via an online questionnaire from patients tested in the programme who actively opt-in to this, and the data are linked to their test result and demographic details [16]. Symptom reporting by patients in CARI is subjective as there are no set clinical criteria.

The first season for the CARI surveillance programme was from 1 November 2021 to 2 October 2022. During this period, there were still some restrictions in certain areas of Scotland on patients presenting in person at GP practices with ARI symptoms, and some patients were still attending community-based COVID-19 Clinical Assessment Centres (CACs) until these were closed in April 2022. After the initial 2021/22 season, the CARI programme was relaunched on 3 October 2022 for the 2022/23 season. For the current study, CARI data were retrieved for the full 2021/22 and 2022/23 seasons (1 November 2021–1 October 2023).

### Statistical methods

For non-sentinel surveillance, rates of positive tests for HPIV reported in ECOSS were determined and stratified by source (i.e. community-based or hospital). As negative tests are not registered in ECOSS, the total number of tests carried out was not known and therefore the latest Scotland mid-year population estimates from the National Records of Scotland (from 30 June 2021) were used as the denominator [17]. After November 2021, HPIV test results from the CARI sentinel programme were identified and extracted separately and therefore excluded from the calculation of HPIV non-sentinel rates. However, the likely very small number of positive HPIV test results from the sentinel GP swabbing programme before the COVID-19 pandemic could not be identified and excluded from the HPIV non-sentinel rates.

Test (or swab) positivity for HPIV over time was determined using CARI sentinel data. This is the total number of swabs positive for HPIV as a proportion of all swabs. Wilson score interval was used to calculate 95% confidence intervals (CIs). Additional analyses were carried out on the CARI data to examine the relationship between numbers of positive HPIV samples and different age/sex groups (male/female 0–4 years, 5–17 years, 18–44 years, 45–64 years and 65 years and older) To then determine the associations between specific symptoms and test results for parainfluenza, unadjusted odds ratios (OR) with 95% CIs were calculated using univariate binary logistic regression analysis. To secure the highest possible quality of data, symptom data were only analysed for the 2022/23 season as the data were not sufficiently complete before this. Not all patients had symptom data, but to evaluate the representativeness of the sample, demographic factors (age and sex) were compared between patients who had symptom data available and patients who did not. All analyses were carried out using a combination of Microsoft Excel and R software version 4.1.2 [18].

## Results

### Electronic communication of surveillance in Scotland (ECOSS) non-sentinel surveillance

**Figure 1:**
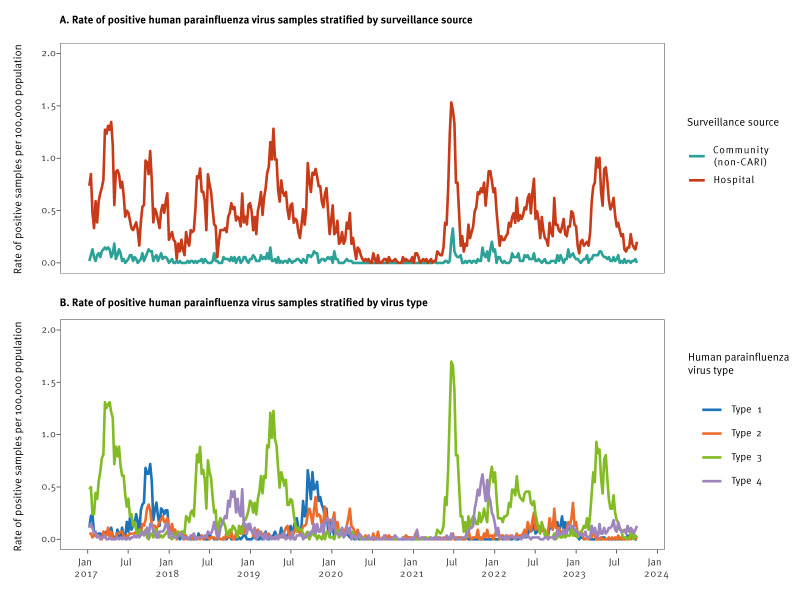
Rates of positive human parainfluenza virus samples from tests undertaken in the community and in hospitals (non-sentinel sources, Electronic Communication of Surveillance in Scotland) stratified by (A) surveillance source and (B) human parainfluenza virus type, Scotland, January 2017–October 2023

Overall, for this period, there were 8,942 HPIV positive samples recorded (8,273 hospital-based and 669 community-based), with 4,344 positive samples in women. The age distribution of positive samples for 0–4 years, 5–17 years, 18–44 years, 45–64 years and 65 years and older was 4,435 (49.6%), 955 (10.7%), 651 (17.7%), 1,075 (12.0%) and 1,826 (20.4%), respectively. Of all the positive samples, 2,638 were recorded in ECOSS (2,401 hospital-based and 237 community-based) during the time period when the CARI programme was operating (between 1 November 2021 and 1 October 2023). Figure 1 shows rates of positive HPIV samples from tests undertaken in the community and in hospitals (non-sentinel sources, ECOSS) in Scotland between January 2017 and October 2023, stratified by surveillance source (Figure 1A) and by HPIV type (Figure 1B).

CARI: the Community Acute Respiratory Infection sentinel surveillance programme.

For hospital-based data, fairly regular peaks of HPIV occurred from January 2017 up to spring 2020, with the highest weekly rates at 1.27 per 100,000 population in March 2017 (69 cases), and 1.28 per 100,000 population in April 2019 (70 cases) (Figure 1A). This was followed by consistent detection of less than 0.10 per 100,000 population after the start of the pandemic in 2020. From summer 2021, there were again regular peaks, with a high of 1.53 per 100,000 population in June 2021 (84 cases). For community-based samples, the positivity rates were even lower and less distinct. However, some similar patterns were seen with weekly peaks of 10 cases in April 2017 (0.18/100,000 population), 8 cases in March 2019 (0.15/100,000 population) and 18 cases in June 2021 (0.33/100,000 population), respectively.

Rates of HPIV-1 reached weekly rates of 0.72 per 100,000 population in October 2017 (39 cases) and 0.66 per 100,000 population in September 2019 (36 cases) (Figure 1B) showing alternate year peaks. Although there were some detections in 2022, alternate year peaks were not observed after 2021. Rates of HPIV-2 followed a similar pattern to rates of HPIV-1, reaching weekly rates of 0.33 per 100,000 population (18 cases) in October 2017 and 0.40 per 100,000 population (22 cases) in October 2019, but again, seasonality was not clearly re-established after the height of the pandemic. The spring peaks in HPIV-3 were clear between 2017 and 2019, with weekly highs of 1.3 per 100,000 population (70 cases) in March–April 2017, 0.88 per 100,000 population (48 cases) in May 2018 and 1.23 per 100,000 population (67 cases) in April 2019. There was then virtually no detection after the start of the pandemic in 2020. In summer 2021, there was a peak in HPIV-3, reaching 1.70 per 100,000 population (93 cases) in June, and rates did not return to the lowest levels before a new peak reached 0.70 per 100,000 population (38 cases) in December 2021. Rates of at least 0.16 per 100,000 population (9 cases per week) continued into the first half of 2022. In 2023, the HPIV-3 peak during the first half of the year was similar in timing and magnitude to the HPIV-3 peaks in 2017 and 2019, reaching a weekly peak of 0.93 per 100,000 population (51 cases) in April 2023, which may represent a return to annual spring peaks. Human parainfluenza virus 4 tended to be more common in the second half of the year throughout the study period, but with virtually no detection after the start of the pandemic in 2020. The highest peak was then recorded in the latter half of 2021, at a weekly peak of 0.62 per 100,000 population (34 cases) in November, but detections have been lower since.

### Community acute respiratory infection sentinel (CARI) surveillance

Between 1 November 2021 and 1 October 2023, 19,341 samples were tested in the CARI surveillance programme (3,900 and 15,441 samples for the 2021/22 and 2022/23 seasons, respectively). There was a total of 975 samples positive for HPIV (249 and 726 for the 2021/22 and 2022/23 seasons, respectively), giving an overall swab positivity of 5.0%.

There were 11,965 (61.9%) tests from females, of which 601 (61.6%) were positive for HPIV. The age distribution of overall samples for 0–4 years, 5–17 years, 18–44 years, 45–64 years and 65 years and above was 2,192 (11.3%), 2,972 (15.4%), 6,494 (33.6%), 4,480 (23.2%) and 3,196 (16.5%), respectively. For samples positive for HPIV, the distribution was 193 (19.8%), 128 (13.1%), 280 (28.7%), 210 (21.5%) and 164 (16.8%), respectively, with a much higher proportion of children aged 0–4 years.

**Figure 2:**
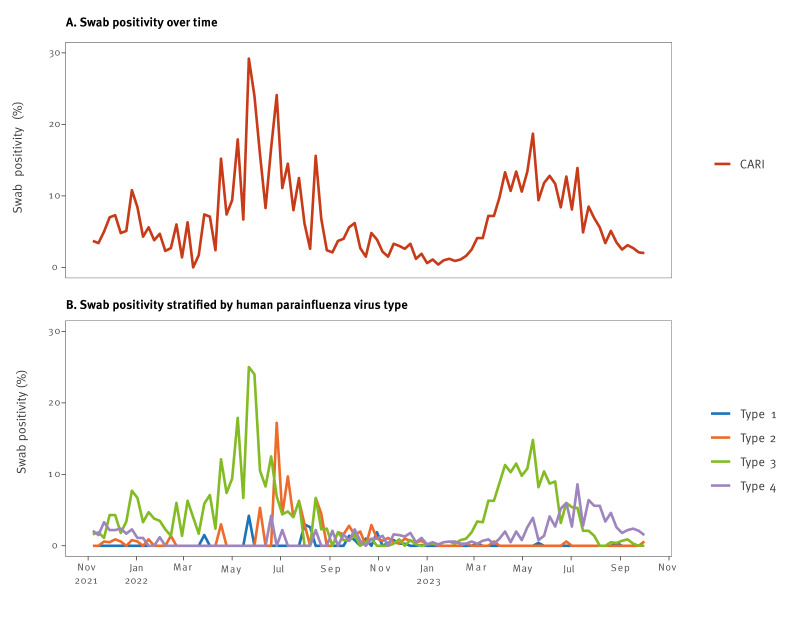
Overall human parainfluenza virus swab positivity in the Community Acute Respiratory Infection (CARI) sentinel surveillance programme (A) over time and (B) stratified by human parainfluenza virus type, Scotland, November 2021–October 2023 (2021/22 and 2022/23)

Figure 2 shows overall HPIV swab positivity in the CARI sentinel surveillance programme over time between November 2021 and October 2023 (the 2021/22 and 2022/23 seasons), stratified by HPIV type. There were some peaks in CARI swab positivity, reaching 10.8% (95% CI: 6.5–17.3) in December 2021; 29.2% (95% CI: 14.9–49.2) in May 2022; and 18.7% (95% CI: 14.0–24.6) in May 2023 (Figure 2A).

Swab positivity is total number of swabs positive for HPIV as a proportion of all swabs.

CARI swab positivity by HPIV type mirrors the hospital and community patterns in Figure 1B fairly well (Figure 2B). However, there was a peak in HPIV-2 swab positivity in June 2022, at 17.2% (95% CI: 7.6–34.5), which was less clear in the non-sentinel hospital and community data. Further, a peak in HPIV-4 swab positivity was seen in May–September 2023, reaching 8.6% (95% CI: 5.1–14.2) in July, which was also not reflected in non-sentinel sources. There was a clear peak during spring 2023, particularly in HPIV-3, with swab positivity reaching 14.8% (95% CI: 10.6–20.3) in May. Overall, HPIV-3 was the predominant type, followed by HPIV-4.

**Figure 3:**
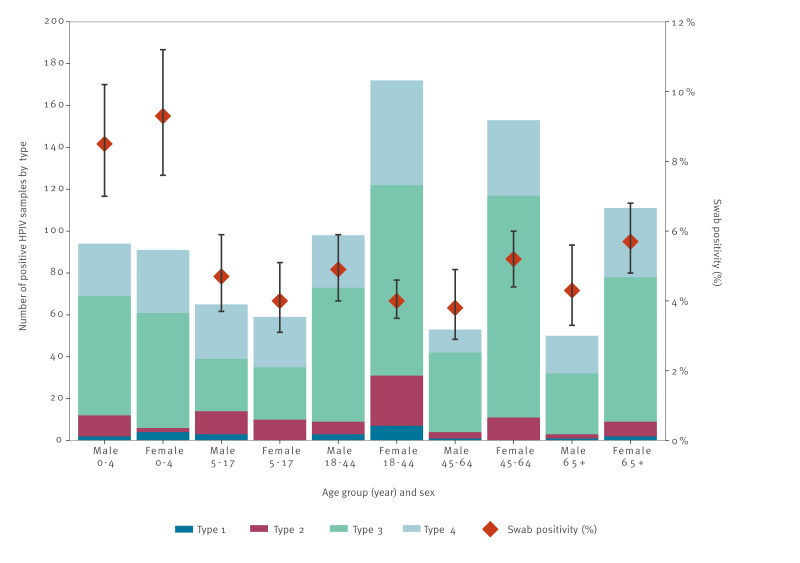
Human parainfluenza virus swab positivity in the Community Acute Respiratory Infection (CARI) surveillance programme and number of positive human parainfluenza virus samples by type, stratified by age and sex, Scotland, November 2021–October 2023 (2021/22 and 2022/23)

Figure 3 shows HPIV swab positivity in the CARI surveillance programme and number of positive HPIV samples by type, stratified by different age/sex groups. The highest number of positive samples was in females aged 18–44 years, followed by females aged 45–64 years. Swab positivity was significantly higher in the 0–4 age group (8.9%, 95% CI: 7.7–10.2) compared with other age groups. Swab positivity in the other age groups ranged from 4.3% in the 5–17 and 18–44 age groups (95% CI: 3.6–5.1, and 3.8–4.8, respectively) to 5.2% in the 65 years and over age group (95% CI: 4.5–6.0). There was no significant difference in swab positivity between females and males in any age group.

HPIV: human parainfluenza virus.

**Figure 4:**
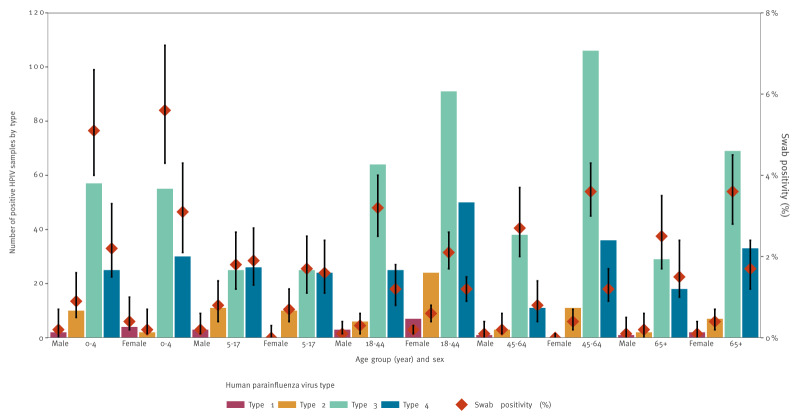
Human parainfluenza virus swab positivity in the Community Acute Respiratory Infection (CARI) surveillance programme and number of positive human parainfluenza virus samples, stratified by HPIV type, age and sex, Scotland, November 2021–October 2023 (2021/22 and 2022/23)

Figure 4 shows CARI swab positivity for HPIV, stratified by HPIV type and different age/sex groups. No significant differences in swab positivity by HPIV type was observed between females and males in any age group. However, the 0–4 age group had a significantly higher swab positivity for HPIV-3 compared with all other age groups, at 5.4% (95% CI: 4.5–6.4). There were no significant differences in swab positivity between age groups for HPIV-1, HPIV-2 and HPIV-4.

HPIV: human parainfluenza virus.

### Symptoms in HPIV-positive patients

The CARI surveillance programme was the only surveillance source with available data on symptoms. Symptom data were available for 209 (31.5%) of HPIV-positive patients in the 2022/2023 season. The most common symptoms were cough (90.9%, 190 cases), runny/blocked nose (76,6%, 160 cases), sore throat (76.1%, 159 cases) and fatigue (75.6%, 158 cases). Further analysis showed that these symptoms were the most common for all four types of HPIV (data not shown). Patients who tested positive for HPIV had a statistically higher unadjusted OR of having a cough (OR: 1.64, 95% CI: 1.06–2.71), a runny/blocked nose (OR: 1.57, 95% CI :1.14–2.21) and sneezing (OR: 1.34, 95% CI: 1.01–1.77), compared with other patients recruited and tested in the CARI surveillance programme. Chills and limb/joint pain were negatively associated with HPIV. Supplementary Figure S1 shows further details on the distribution of symptoms in patients positive for HPIV in the CARI surveillance programme in 2022/23. It also depicts the unadjusted OR with 95% CI for each of the reported symptoms in patients positive for HPIV in the CARI surveillance programme in 2022/23, compared with other patients recruited and tested in the CARI surveillance programme.

## Discussion

The COVID-19 pandemic caused changes in circulation patterns of respiratory virus infections [19]. This study explored epidemiological and clinical patterns of HPIV before and after the COVID-19 pandemic in Scotland. Pre-pandemic, HPIV followed typical seasonal trends [4-7]. However, from spring 2020 to spring 2021, the number of HPIV-positive tests decreased due to strict lock-downs and strengthened infection prevention and control measures, which reduced both exposure to viruses and community testing [20,21]. From spring 2021, HPIV positivity increased sharply, suggesting a return to pre-pandemic levels as restrictions on social distancing were lifted. However, the seasonal patterns of HPV-1 in particular seem disrupted, and alternate-year peaks are not yet re-established. Similar patterns of HPIV fluctuation related to the COVID-19 pandemic have been observed in other countries, indicating a global phenomenon influenced by public health measures and changes in social behaviour during the pandemic [9,10].

In general, results from non-sentinel and sentinel surveillance of HPIV were comparable, with some small differences. For example, the CARI surveillance programme identified peaks of HPIV-2 and HPIV-4 that were not seen in routine ECOSS non-sentinel data. Since hospitals contributed considerably more samples to the ECOSS data than community settings did, due to primary care patients generally being less severely ill and precise pathogen identification being less critical for treatment decisions, the CARI programme is more likely to identify peaks of circulating pathogens with less severe clinical outcomes.

Differences in demographic profiles (e.g. age and sex) of patients in different surveillance systems are common since a surveillance system focused on hospitalised patients will inherently have different, and likely more severe, positive cases compared with a community surveillance programme like CARI [16]. Various systems can be interpreted together to provide a more comprehensive understanding of a disease, even though they do not always represent the exact same patient population. This increased awareness of respiratory viral pathogens circulating in the community at unusually high levels, along with insights into community-level dynamics, may alert primary care clinicians to be more vigilant, particularly with young or immunosuppressed patients. This has important value from a public health perspective.

Human parainfluenza virus is in general most common in children under 5 years [7]. This was supported by findings in the present study, with the highest HPIV swab positivity in this age group, while swab positivity was broadly similar for all other age groups. Between 1998 and 2013 in England, differences in HPIV swab positivity between different age groups became smaller over time, with a decline in detection in very young children (< 1 year) and an increase in people aged over 65 years [4]. Although the highest absolute numbers of positive samples in the CARI surveillance programme in our study were from females in the 18–44 age group followed by females in the 45–64 age group, this was because more women in these age groups were recruited to the programme compared with other age/sex groups (data not shown) and does not indicate higher prevalence.

It is also acknowledged that symptom reporting by patients is subjective and depends on individual circumstances. Three of the four symptoms most commonly reported are included in the CARI case definition, and so patients were included in the CARI programme and tested because they presented with these symptoms. However, the symptoms cough, runny/blocked nose and sneezing were more likely to be associated with patients who were positive for HPIV compared with other patients recruited and tested in the CARI surveillance programme. The additional symptom surveillance adds value to the programme, enabling examination of the associations between clinical symptoms and specific respiratory pathogens, which can also be used for monitoring change in patterns of symptoms over time. Combining different sources of surveillance data that have different strengths and weaknesses is likely to give a more comprehensive picture of disease patterns than relying on one system alone [22].

This study demonstrates the value of combining information from different surveillance systems, although CARI surveillance only started in November 2022. There are other limitations associated with this study. During the peak of the pandemic as well as at the onset, the existence of public health guidance and government policies in the UK restricted movement of individuals and their ability to present at a GP practice. This will have decreased the numbers of patients diagnosed with HPIV in the community as well as in the CARI surveillance programme. Further, swab positivity for HPIV, the primary outcome for CARI surveillance, is the number of positive samples divided by the total number of samples collected. Testing for 10 pathogens means that the prevalence of other pathogens can affect this rate. High rates of other pathogens can increase the total number of tests, potentially reducing HPIV swab positivity. However, this effect is likely to be small. As with any surveillance programme, the CARI data may not give a completely accurate picture of respiratory pathogens in the community setting as not all patients with ARI present to their GP, and the CARI case definition does not necessarily identify all those that do.

## Conclusion

This study describes patterns of HPIV detection in Scotland before and after the COVID-19 pandemic. The decline during the peak of the pandemic and at least partial resurgence thereafter is likely to be the result of complex interplay between viral epidemiology and public health measures. The combination of different surveillance systems helps provide a better understanding of HPIV dynamics and shows the importance of integrating different data sources for a nuanced assessment. Insights into age-specific and symptom-associated patterns contribute to our understanding of HPIV epidemiology and can inform public health strategies, such as promoting awareness, fostering collaboration to mitigate the impact of HPIV infections, implementing infection control measures and stimulating research into vaccine development.

## Supplementary Data

225000 Supplementary Figure S1
