# Supplementary material for: Trends in human parainfluenza virus in Scotland before and after the peak of the COVID-19 pandemic, January 2017 to October 2023
Source: Euro Surveill. 2025 Jan 16;30(2):2400147. doi: 10.2807/1560-7917.ES.2025.30.2.2400147 (PMC11740291; doi:10.2807/1560-7917.ES.2025.30.2.2400147)
Supplement: Supplementary Figure S1 [file 24-00147_LAIRD_SupplementaryFigureS1.pdf]

This supplementary material is hosted by *Eurosurveillance* as supporting information alongside the article' Trends in human parainfluenza virus in Scotland before and after the peak of the COVID-19 pandemic, January 2017 to October 2023', on behalf of the authors, who remain responsible for the accuracy and appropriateness of the content. The same standards for ethics, copyright, attributions and permissions as for the article apply. Supplements are not edited by *Eurosurveillance* and the journal is not responsible for the maintenance of any links or email addresses provided therein.

**Supplementary Figure S1**

**The distribution of symptoms in patients positive for HPIV in the CARI surveillance programme in 2022/23. Unadjusted OR with 95% CI for each of the reported symptoms in patients positive for HPIV in the CARI surveillance programme in 2022/23, compared to other patients recruited and tested in the CARI surveillance programme.**

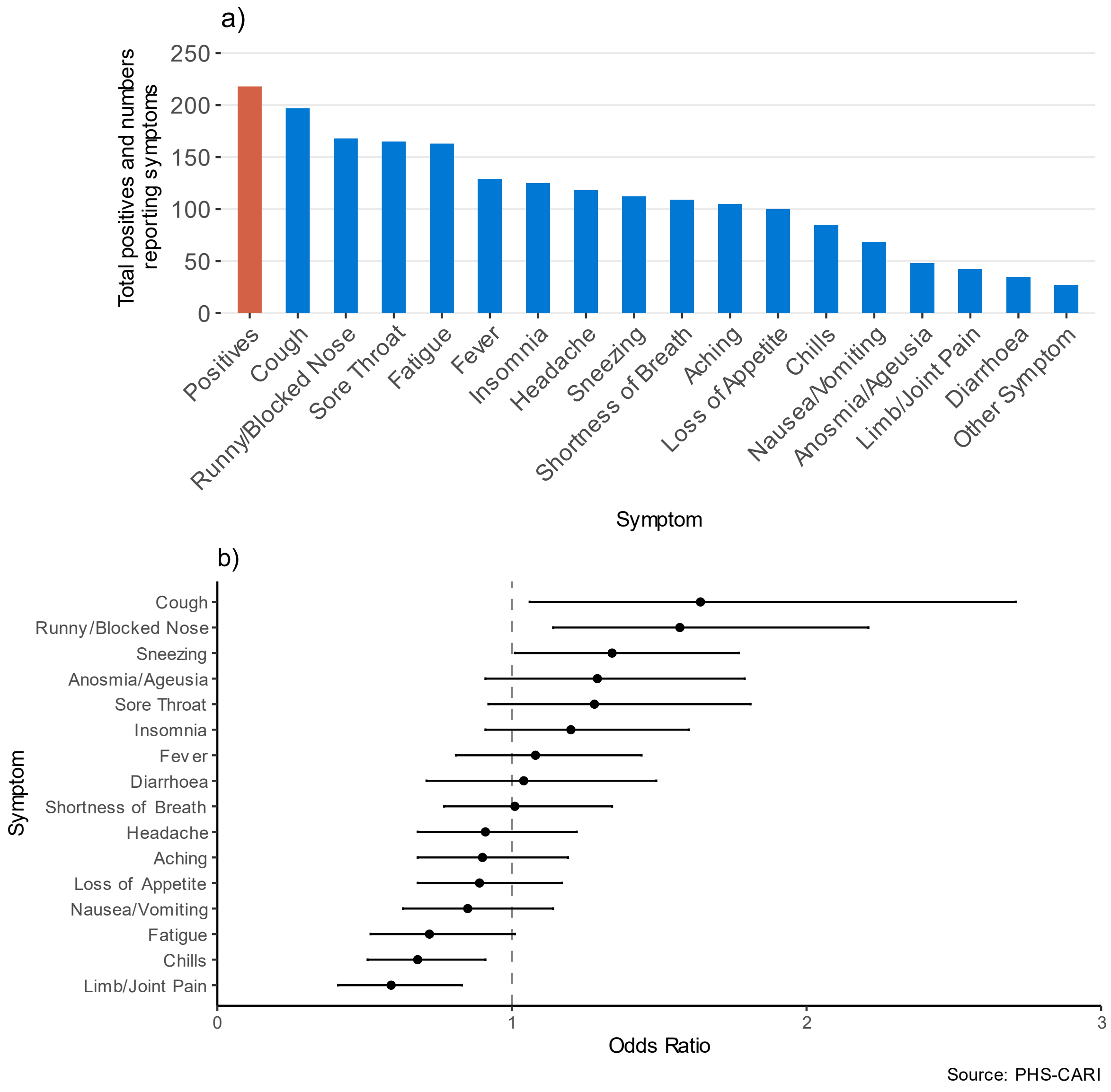


Symptom reporting by patients in CARI is subjective; there is no set clinical criteria
